# Survey on Visual Impairment and Refractive Errors on Ta’u Island, American Samoa

**Published:** 2011-01

**Authors:** Shawn S Barnes, Pamela-Jaimelyn M Utu, Lauren Sumida, Darragh C O’Carroll, Tyrie L Jenkins, John Corboy

**Affiliations:** 1 Outbound Eye Health International, Honolulu, Hawaii, USA; 2 John A. Burns School of Medicine, University of Hawaii, Honolulu, Hawaii, USA; 3 Tyrie Lee Jenkins, MD (Laser Eye Center of Hawaii) Inc., Honolulu, Hawaii, USA; 4 Hawaiian Eye Foundation, Honolulu, Hawaii, USA

**Keywords:** American Samoa, Refractive Error, Visual Impairment, Visual Acuity, Rural

## Abstract

**Purpose:**

To assess the prevalence of presenting visual impairment and refractive errors on the isolated island of Ta’u, American Samoa.

**Methods:**

Presenting visual acuity and refractive errors of 124 adults over 40 years of age (55 male and 69 female) were measured using the Snellen chart and an autorefractometer. This sample represented over 50% of the island’s eligible population.

**Results:**

In this survey, all presenting visual acuity (VA) was uncorrected. Of the included sample, 10.5% presented with visual impairment (visual acuity lower than 6/18, but equal to or better than 3/60 in the better eye) and 4.8% presented with VA worse than 6/60 in the better eye. Overall, 4.0% of subjects presented with hyperopia (+3 D or more), 3.2% were myopic (−1 D or less), and 0.8% presented with high myopia (−5 D or less). There was no significant difference between genders in terms of visual impairment or refractive errors.

**Conclusion:**

This study represents the first population-based survey on presenting visual acuity and refractive errors in American Samoa. In addition to providing baseline data on vision and refractive errors, we found that the prevalence of myopia and hyperopia was much lower than expected.

## INTRODUCTION

While a wealth of population-based data on visual impairment and refractive errors exists for the Asia-Pacific region,^1^–^4^ the vast majority of these reports are confined to Asia. The Pacific region is often neglected in terms of population-based ocular surveys; notable exceptions include Tonga^5^, Cook Islands^6^, and Vanuatu^7^. The central Pacific islands of Samoa and American Samoa have been the subject of previous prevalence studies on various ophthalmic conditions,^8^–^10^ however, the study populations in these reports have been either solely clinic-based or obtained through chart review. Herein, we report the first population-based prevalence survey of presenting visual acuity and refractive errors in the Samoan archipelago. Through this small-scale study on a remote island, our hope is to establish baseline data and to encourage further and more robust epidemiological work on eye health in both urban and rural settings in Samoa.

**Figure 1:**
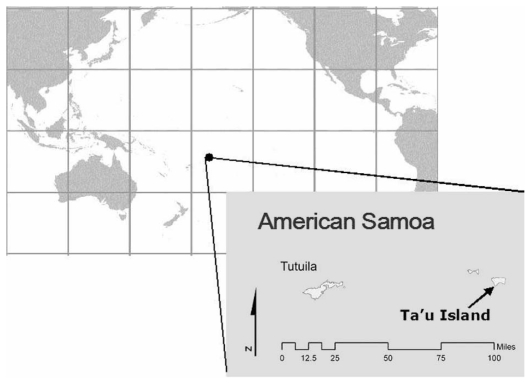
Location of Ta’u Island, American Samoa.

The Samoan archipelago is located approximately midway (~4,000 km) between the Hawaiian Islands and Australia (Fig 1). While often thought of as one entity, there are two Samoas, the Independent Nation of Samoa and American Samoa. Archaeological evidence have suggested the presence of humans in the Samoan archipelago 3,000 years ago.^11^ The archipelago was politically split in 1878, when the United States annexed the islands of Tutuila, Ofu, Olosega, and Ta’u, which became the U.S. territory of American Samoa.

Ta’u Island is approximately 44 km^2^ in area and located 100 km east of the main island of Tutuila. The approximately 850 residents of Ta’u, while a part of the American social services system, tend to practice more of a subsistence or traditional lifestyle as compared to inhabitants on the main island of Tutuila. In the year 2000, 27.7% of Ta’u island’s inhabitants were 40 years of age or older and male subjects comprised 51.1% of the population. Ta’u is perhaps most famous for being the site of Margaret Mead’s anthropological research in the 1920’s.^12^ There is one practicing ophthalmologist in American Samoa at Lyndon Baines Johnson Hospital on Tutuila. However, before the arrival of an emergency medicine doctor in 2009, there had been no resident physicians on Ta’u.

## METHODS

In the summer of 2009, eye-screening events were held in the villages of Ta’u, Fitiuta, and Faleasao on the island of Ta’u, American Samoa. These events were held as part of a community outreach program in coordination with the University of Hawaii, School of Medicine and the non-profit organization, Outbound Eye Health International. Events were performed at village community meeting locations in each village. Permission to hold screening events was given by both the American Samoa Department of Health Independent Review Board (IRB) and the local mayor of each village. Informed consent was obtained from all participants and guidelines of the Declaration of Helsinki were adhered to. All residents of Ta’u Island over the age of 40 were invited to participate in the screening program, comprising a convenience sampling of the island. Of 242 people over the age of 40 on Ta’u island^13^, 124 participated (51.2% participation rate). All these residents cooperated and fully participated in the study. The average age of participants was 56.7±11.1 years and 44.4% of the participants were male (Table 1).

Presenting visual acuity was assessed by a standard Snellen chart, in controlled indoor lighting conditions at 6 meters. Best-corrected visual acuity was not assessed. Non-cycloplegic refractive error was measured using a Canon R-22 autorefractometer (Canon Inc., Tokyo, Japan). Following examination, results for each participant were explained in local language and each participant was provided with a complimentary pair of sunglasses and reading glasses. Any identified pathology was referred to the ophthalmologist at the Lyndon Baines Johnson hospital on Tutuila.

Presenting visual acuity was defined as visual acuity measured at the time the participant presented to the survey: either unaided, or with spectacles if worn. No participant in this study presented with spectacles, as refractive correction services are very limited in the territory. Presenting visual impairment was defined as presenting visual acuity worse than 6/18 in the better eye, i.e. the combination of low vision (defined by the WHO as best-corrected acuity between 6/18 and 3/60) and functional blindness (defined by the WHO as best-corrected acuity worse than 3/60).^14^

Refractive error was categorized following the methods of the Eye Diseases Prevalence Research Group.^15^ Hyperopia was defined as a spherical equivalent of +3 D or more. Myopia was defined as spherical equivalent of −1 D or more, and high myopia was defined as spherical equivalent exceeding −5 D.

## RESULTS

Table 1 shows presenting visual acuity and refractive error data.

### Visual Acuity

Of 124 participants, 13 (10.5%) presented with visual impairment and 6 (4.8%) presented with visual acuity less than 6/60. There was no statistical difference in presenting visual impairment (P=0.455) or VA equal to or worse than 6/60 (P=0.892) between genders.

### Refractive Errors

Of 124 participants, 5 (4.0%) were hyperopic, 4 (3.2%) were myopic, and 1 (0.8%) was highly myopic. Of 55 male subjects, 1 (1.8%) was hyperopic, 2 (3.6%) were myopic, and 1 (1.8%) was highly myopic. Of 69 female subjects, 4 (5.8%) were hyperopic, 2 (2.9%) were myopic, and none were high myopes. There was no statistical difference between genders in hyperopia (P=0.509), myopia (P=0.780), or high myopia (P=0.115). Average spherical equivalent refractive error for all participants was +0.35D.

## DISCUSSION

Data on presenting visual impairment are often not reported in eye surveys. Given minimal access to refractive correction services in this particular study (none of the 124 participants used spectacles), presenting vision may be of more practical importance as compared to best-corrected visual acuity. Compounding this, Samoan attitudes towards disease have been reported to be significantly fatalistic, perhaps leading to a tendency to view eye disease as a natural component of aging and avoidance in seeking care.^16^

Refractive errors on Ta’u island were surprisingly uncommon. The prevalence of myopia (−1.0 D or less) reported here (3.2%) is similar to clinic-based prevalence rates previously reported for Samoa^8^,^9^, but significantly lower than that reported in Australia^17^ (12.6%) and the USA^18^ (16.8%). Speculations on the cause of this difference may include genetic (such as a history of population bottlenecks) or environmental factors (such as the relative lack of near-work in rural Samoa).^19^ Similarly, the hyperopia (+3 D or higher) rate (4.0%) was significantly lower than Australia^17^ (7.7%) and the USA^18^ (9.8%). Such low rates of refractive errors and high rates of low visual acuity suggest that visual impairment on Ta’u island is due to ocular morbidity rather than refractive errors which should be further assessed.

This study represents the first population-based survey on visual acuity and refractive errors in Samoa. However, it should certainly not be the last. The limited sample size, limited geographic area, and lack of best-corrected visual acuity data in this study calls for further work in establishing the prevalence and causes of ocular morbidity in Samoa. In addition, emphasis should be placed on sampling both urban and rural Samoan populations, because lifestyle, culture, and access to care can be significantly different between these environments. Urban and rural prevalence data would be useful in both delineating demographic trends and proper delivery of eye care in American Samoa.

## Figures and Tables

**Table 1:** Presenting visual impairment and refractive errors among adults 40 years and older on Ta’u Island, American Samoa

Groups	Number (%)	Presenting visual impairment*	VA worse than 6/60	Hyperopia*	Myopia*	High Myopia*
40 to 59 years	77 (62.1%)	3 (4.0%)	2 (2.6%)	1 (1.3%)	1 (1.3%)	0
≥60 years	47 (37.9%)	10 (21.3%)	4 (8.5%)	4 (8.5%)	3 (6.4%)	1 (2.1%)
Total	124 (100.0%)	13 (10.5%)	6 (4.8%)	5 (4.0%)	4 (3.2%)	1 (0.8%)

Male	55 (44.4%)	4 (7.3%)	2 (3.6%)	1 (1.8%)	2 (3.6%)	1 (1.8%)
Female	69 (55.6%)	9 (13.0%)	4 (5.8%)	4 (5.8%)	2 (2.9%)	0

VA, visual acuity

* Presenting visual impairment was defined as presenting visual acuity of less than 6/18, but equal to or better than 3/60 in the better eye; hyperopia was defined as spherical equivalent greater than +3 D; myopia as spherical equivalent exceeding −1 D; and high myopia as spherical equivalent of −5 D or more.
